# Tertiary Lymphoid Structures: An Anti-tumor School for Adaptive Immune Cells and an Antibody Factory to Fight Cancer?

**DOI:** 10.3389/fimmu.2017.00830

**Published:** 2017-07-21

**Authors:** Jean-Luc Teillaud, Marie-Caroline Dieu-Nosjean

**Affiliations:** ^1^INSERM, UMRS 1138, Cordeliers Research Center, Team “Cancer, Immune Control and Escape”, Paris, France; ^2^Paris Descartes University, Sorbonne Paris Cite, UMRS 1138, Cordeliers Research Center, Paris, France; ^3^Pierre and Marie Curie University (UPMC), Paris 06, Sorbonne University, UMRS 1138, Cordeliers Research Center, Paris, France

**Keywords:** tertiary lymphoid structure, B cell, tumor immunity, plasma cell, cancer patient, T cell, antibody, immune checkpoint

## Abstract

Tertiary lymphoid structures (TLS) present in human solid tumors are essential for the shaping of a favorable immune micro-environment to control tumor development in most cases. They represent a formidable school for T-cell priming, B cell activation, and differentiation into plasma cells and an exquisitely located factory for antibody production. The manipulation of TLS neogenesis and maintenance represents, therefore, an exciting task to set up efficient anti-cancer vaccine strategies leading to long-lasting anti-tumor adaptive responses. To achieve this goal, a number of important issues are still pending. How TLS-T and -B cells and antibodies locally produced are related to the improved survival of cancer patients with high density of TLS is still unclear. In addition, the mechanisms by which tumors escape the immune surveillance exerted by TLS are still poorly understood and the role of immune suppressive cytokines, regulatory T cells, and/or antibodies in this process remains to be explored. The identification of the key parameters that distinguish TLS with anti- or possible pro-tumor activity is also essential to make the therapeutic targeting of TLS a success. Finally, how TLS-based therapeutic approaches can be associated with targeted therapies or immunointerventions, such as the use of ICP blockers to improve anti-tumor responses, is an open question. We will discuss these different issues in the present review.

## Introduction

In 2008, it was shown that lung tumors exhibit tertiary lymphoid structures (TLS) (also termed at that time tumor-induced bronchus-associated lymphoid tissues) and that these structures correlate with a favorable clinical outcome in non-small-cell lung cancer (NSCLC) patients (1). Since then, these observations have been extended to a large number of other tumor types, such as breast cancer, colorectal cancer, head and neck carcinoma, and melanoma [reviewed in Ref. (2)]. Several studies have contributed to our understanding of the local anti-tumor immune responses that arise in the close vicinity of tumor masses, if not within (3–7). Interestingly, tumor-associated TLS exhibit strong similarities with lymph node (LN) organization. TLS is mostly composed of T cells and of mature dendritic cells (DC) located in the T cell rich areas closed to a B-cell follicle. These segregated zones are suggestive of the setting of a local antigen (Ag)-driven antibody response, which could lead to the production of antibodies with anti- or pro-tumor properties. Moreover, TLS-B cell zone also present features of LN-B cell follicle marked by the homing of both naïve and germinal center (GC) B cells, with scattered plasmablasts and memory B cells. B cells are also Ag-presenting cells (APC) that can recruit and activate T cells, in a cognate or non-cognate manner, and trigger T-cell polarization, therefore impacting positively or negatively T-cell-mediated anti-tumor responses. Thus, in the present review, we will summarize and discuss the role of tumor-infiltrating B cells associated with TLS and their possible impact on patient outcome, as the prognosis value of these cells remains debated. Since TLS have been mostly associated with a better prognosis of cancer patients [reviewed in Ref. (2)], we will also discuss the development of immunotherapeutic strategies aimed at manipulating TLS to strengthen adaptive anti-tumor responses. Finally, we will present how biomarkers related to TLS might be useful for the follow-up of patients under therapy.

## TLS as a Local Antibody Factory

As in secondary lymphoid organs, GC found in TLS contain proliferating B lymphocytes that express the enzyme activation-induced deaminase (AID), central to class switch recombination (CSR) and somatic hypermutation (SHM) processes that ultimately lead to the production of high-affinity antibodies other than IgM. TLS B cell follicles also include mesenchymal follicular dendritic cells (FDC), tingible-body macrophages that phagocytize apoptotic non-selected B cells, follicular helper T cells (T_FH_) required for GC formation and B cell differentiation, as well as plasmablasts undergoing their terminal development into cells secreting large amounts of high-affinity antibodies. Notably, tumor-infiltrating B lymphocytes have a limited usage of immunoglobulin (Ig) variable genes and are submitted to CSR and SHM events. Thus, it has been suggested that an Ag-driven antibody response occurs in TLS in different tumors (breast medullary and breast ductal carcinoma, metastatic melanoma). In particular, we have demonstrated that proliferating GC-B cells expressing AID and surrounded by FDC and T_FH_ cells highlight an ongoing immune response taking place in NSCLC-associated TLS. Taking into account that an elevated number of TLS-B cells correlates with the long-term survival of NSCLC patients, we speculated that TLS represent a critical site for the development of an efficient B-cell-dependent anti-tumor immunity (5). It has been suggested that TLS-associated antibody responses may be elicited by tumor-associated antigens (TAAs) or self-Ags (8). Recently, we have shown that about 50% of the NSCLC tumors with a high density of TLS exhibit B lymphocytes that produce antibodies to at least one lung TAAs tested (e.g., LAGE-1, MAGE-A1, MAGE-A4, P53, and NY-ESO1), already described to induce endogeneous anti-tumor immunity in patients (5). One difficulty in assessing the humoral response associated with TLS is to screen for antibodies capable of binding molecules other than proteins, such as glycolipids, lipids, gangliosides or sugars. These screenings require the handling of more complex techniques. For instance, the specificity of antibodies produced in the tumor micro-environment has been investigated using antibody phage display (9, 10) or a combination of cellular and molecular techniques based on the isolation of B cells/plasma cells followed by the cloning of VH and VL domains. However, such screenings are severely hampered by the limited amounts of purified molecules to be investigated as putative Ags. Interestingly, antibodies directed against non-protein tumor-associated carbohydrate Ags [i.e., disialoganglioside GD3 in medullary breast carcinoma (10)] have been isolated from phage libraries generated from tumor-infiltrating B cells, suggesting that a number of tumor specificities lies in molecules other than proteins (11). Moreover, it is still unclear whether the antibodies produced by plasma cells infiltrating tumors bind Ags and epitopes identical to those recognized by antibodies produced at distant sites (e.g., bone marrow, LNs, and spleen). In a pilot study conducted in NSCLC, the reactivity of tumor-specific antibodies secreted by tumor-infiltrating plasma cells rarely overlapped with the reactivity observed in the sera of patients (5). The emergence of specific neo-Ags as a result of mutations in tumor cells could be also a strong trigger for the rapid and local production of specific antibodies, besides the shared TAA identified so far. Whether TLS-derived antibodies could represent a particular category of antibodies aimed at fighting the mutation escape process of tumor cells is an important issue that should be addressed, as it is currently investigated for T cells.

Another important issue raised by the *in situ* production of antibodies is the function of these molecules. It remains to be investigated in details. A number of reports have described the production of high-affinity anti-tumor antibodies, mostly IgG, by intra-tumoral B lymphocytes and animal models have made it possible to demonstrate the ability to these antibodies to impact tumor development (12–15). These pre-clinical data have favored the idea that antibodies produced within the tumor masses through the generation of TLS contribute to the anti-tumor response, as already suggested by the correlation between the presence of plasma cells, IgG, or of kappa/lambda chains in tumors, and a more favorable outcome in breast cancer, NSCLC, colorectal cancer, metastatic melanoma, and ovarian cancer patients (16–21). Anti-tumor IgG antibodies produced *in situ* could kill tumor cells through the activation of the complement classical pathway through the bonding of C1q (although the role of complement in the anti-tumor efficacy of a number of therapeutic antibodies has not been firmly established), the induction of programmed cell death, or indirectly by activating immune cells expressing receptors for the Fc region of IgG (FcγRs), leading to the triggering of antibody-dependent cell-mediated cytotoxicity (ADCC) and to the release of cytokines (i.e., IFN-γ and IL-12) that can in turn activate cells from the innate immunity (such as neutrophils, NK cells, macrophages, and DC) and strongly impact T helper (Th) cell polarization. However, there is no definitive evidence in patients that locally produced anti-tumor IgG response can play these functions. Only IgG deposit on cancer cells has been reported in many malignancies so far.

The function of antibodies produced locally and present within the tumor or in its close vicinity is likely heavily dependent on the class of antibodies produced and possibly of their glycosylation pattern. In this respect, one should distinguish IgG subclasses (IgG1 and IgG3) capable of activating the classical pathway of complement through the binding to C1q and of triggering ADCC, from IgG2, IgG4, and IgA. These latter isotypes are produced mostly upon chronic Ag exposure, do not activate the complement cascade (IgG2, IgG4, IgA), and have a poor ability to bind FcγR (IgG2, IgG4). In addition, it has been suggested that these isotypes could be involved in the setting of immune tolerance. Moreover, TGF-β and IL-10, often considered as immune suppressive cytokines and present in the tumor micro-environment, induce IgA and IgG4 switching. Interestingly, it has been suggested that these isotypes are likely to contribute to immunosuppression in cancer patients. Shalapour *et al*. reported that the anti-tumor T cell response triggered by immunogenic chemotherapeutic drugs is strongly down-regulated by the presence of tumor-infiltrating IgA^+^ plasma cells in mice engrafted with prostate tumors (22). Similarly, the analysis of human prostate tumors has shown the presence of IgA^+^ plasma cells in the close vicinity of lymphoid-like structures and a lower B cell/CD8^+^ T cell ratio in patients with a strong IgA^+^ plasma cell infiltrate. Thus, the presence of tumor-infiltrating IgA^+^ plasma cells could be associated with tumor immune escape, at least in some cancer types. Importantly, the study of Ig gene rearrangement in micro-dissected TLS-B cells from metastatic melanoma has indicated the same frequency of IgA and IgG switch events, demonstrating that the production of Ig in TLS is not restricted to that of IgG but also includes that of IgA (3). Of note, a number of cancer patients exhibit also tumor-infiltrating IgA^+^ plasma cells with significant serum levels of IgA directed against TAA. Similarly, high serum IgG4 levels inversely correlate with the survival of melanoma patient (23). Furthermore, the same study showed the presence of plasma cells within the tumor, producing IgG4 directed to cancer cells. Interestingly, these antibodies were unable to trigger tumor cell killing—as it could be expected with IgG4—and inhibited the anti-tumor activity of IgG1, possibly by impacting FcγRI (CD64) function. The presence of IgG4^+^ plasma cells in extrahepatic cholangiosarcoma has been positively and negatively correlated with that of regulatory T cells and CD8^+^ T lymphocytes, respectively. Furthermore, these patients who exhibit IgG4^+^ plasma cells had a poor clinical outcome. Thus, the local production of IgG4 within tumors, eventually promoted by a Th2-oriented inflammation, could participate to the immune escape. Overall, these data suggest that IgG1^+^/IgG3^+^ and IgA/IgG4^+^ plasma cells may exert opposite functions in anti-tumor immunity.

## TLS as a School for Adaptive Immune Cells

On the one hand, antibodies produced locally could also contribute to an efficient priming of T cells with anti-tumor activity. The capture of TAA complexed with locally produced IgG through FcγR expressed on various DC subsets could be followed by TAA-derived peptides MHC-I cross-presentation to naïve CD8^+^ T cells. Two studies that used mouse models underline the essential role of the TAA-antibody complexes for the priming of anti-tumor CD8^+^ T cells through DC (24, 25). In another pre-clinical study, it has been shown that infusion of DC loaded with ovalbumin and antibody immune complexes in C57Bl/6 mice enable to generate a strong anti-tumor immunity specific for tumor cells expressing ovalbumin, while no anti-tumor protection is achieved when FcγR-deficient DC are injected (26). In this experimental setting, the anti-tumor effect was lost when the expression of β2-microglobulin, or transporters associated with Ag processing, or of MHC class II molecules was abrogated in DC, indicating that the uptake of IC through FcγR results in MHC class I- and class II-dependent immunity. Of note, a clinical benefit of exposure to IC has also been observed in cancer patients. Treatment of ovarian cancer patients with a murine Technetium-99m-labeled monoclonal antibody in the presence of circulating tumor Ag CA125 induced specific B- and T-cell responses and a favorable outcome in some patients (27). The blockade of IFN-γ secretion by peripheral blood mononuclear cells stimulated with purified CA125 or autologous tumor using anti-MHC class I and II antibodies suggested that a number of patients had developed specific Th and Tc cells after the radiolabeled antibody.

On the other hand, Ag presentation by B lymphocytes can allow the generation of Ag-specific memory CD4^+^ T cells, as well as their boost upon antigenic recall. B cells that can express co-stimulatory molecules upon activation, may represent the key APC within tumors. TLS exhibit an oligoclonal B cell proliferation of Ag-driven B cells and, hence, it can be suspected that they are enriched B cells with specificity against tumor-associated Ags. Thus, TLS-B lymphocytes could efficiently capture Ag derived from tumor cells through their BCR. The likelihood of an APC role of B cells in TLS has been strengthened by studies that showed that cognate interactions occur between TLS-B cells and activated T cells in melanoma. Strikingly, a better survival of patients exhibiting both TLS B cells and activated T cells was observed in this study (28). Overall, B and Th1/CD8^+^ T cell infiltrates parallel a better outcome in a number of malignancies, as compared to each cell type alone (29–32). In addition, a highest CD4^+^ T cell clonality was observed in tumors having a strong number of TLS-B lymphocytes in NSCLC patients. This strengthens the view that TLS-B cells are strongly involved in the setting of a potent anti-tumor adaptive immune T-cell response (8). The ability to present tumor-derived Ags is not restricted to B cells. IgM^+^ and IgA^+^ plasmablasts that express low amounts of surface Ig could also present TAA in tumor-associated TLS to CD4^+^ T cells (33, 34). B cells and plasmablasts may, therefore, modulate T cell fates within the tumor micro-environment through their ability to present tumor-derived peptides. B lymphocytes could also influence T cell polarization and functions by secreting type 1 (IL-12, IFN-γ), type 2 (IL-4, IL-13), and immune suppressive (IL-10, IL-35, TGF-β) cytokines. Production of IL-12 and IFN-γ by intra-tumoral B lymphocytes in hepatocellular carcinoma indicates a putative role of these cells in the induction and/or the amplification of a strong cellular immune response (30). However, tumor-infiltrating B cells could also act as negative regulators of the anti-tumor response, as already discussed for the role of IgA produced locally (see above). The secretion of IL-10, TGF-β, or granzyme-B by B lymphocytes affects anti-tumor immune response. These cells that are considered as regulatory B cells, are responsible for the expansion of T_reg_ cells, promoting tumor progression. Also in tumors, B cells and plasma cells can strongly express PD1 ligand-1 (PD-L1) and PD-L1-expressing IgA^+^ plasma cells can dampen CD8^+^ T cell-mediated immunity (22, 35, 36). Thus, Ti-TLS B cells, plasma cells, and the locally secreted antibodies impact T cell functions, increasing or down-modulating anti-tumor immune response through their capacity to generate IC, to produce Th cell polarizing cytokines, such as IFN-γ, IL-12, IL-4, or IL-13 or immunosuppressive cytokines, such as IL-10 and TGF-β, and to express ligands for stimulatory or inhibitory immune checkpoints. The possible pro-tumor impact of TLS has been also highlighted by Finkin *et al*. who showed that tumor progenitor cells could niche in TLS of a subset of HCC [mainly HCV^+^, non-alcohol-dependent patients (37)], although this observation has not been reported for other solid tumor types so far.

## TLS and Therapy

Tertiary lymphoid structures present in solid tumors before any immune intervention may help the selection of patients who could respond favorably to immunotherapy protocols. Thus, the quantification of TLS in tumors could be a useful biomarker that will help to define inclusion criteria, with the goal to recall tumor-specific T cells and to boost ongoing anti-tumor adaptive responses. Thus, the capacity of inducing TLS might be highly helpful to cancer patients to build-up a long-lasting anti-tumor immune responses, as suggested in pre-clinical studies. However, a number of questions remain to be answered to make it possible to manipulate TLS in immunotherapeutic protocols. First, one needs to identify the cellular and molecular mechanisms leading to TLS formation in the tumor to set up relevant therapeutic approaches that should be successfully used. Second, it is still unclear which mechanisms occur in TLS that trigger an efficient anti-tumor response as opposed to TLS mechanisms that favor tumor progression. This still needs to be carefully explored. Alongside with this issue, the identification of the key factors that distinguish TLS with anti- or pro-tumor activity should be a major goal to render possible the therapeutic targeting of TLS. Finally, it is essential to define how TLS manipulation could be combined with targeted therapies or immunotherapies currently developed. Notably, ICP neutralization that leads to a significant increase of stable responses and longer survival, but in a limited number of cancer patients, could be associated with TLS manipulation. This could be highly beneficial for patients that are poor responders to ICP blockade, if any, thanks to the development or recall of a strong TLS-dependent adaptive anti-tumor immune response not hampered by ICP. In particular, patients exhibiting metastatic melanoma or melanoma tumors that could not be surgically removed, as well as NSCLC patients who did not respond to chemotherapy have shown a significantly better survival after receiving anti-PD1/PD-L1 treatment. Among them, those exhibiting intra-tumor TLS represent a target of choice for combined therapies (38–42). Furthermore, since neo-Ags arise all along tumor development, mostly through a high rate of somatic point mutations, the *in situ* presence of TLS may help a faster immune adaptive response to this rapidly evolving landscape of immunogenic motifs. This could be proven true with heavy smoker NSCLC patients having a high rate of tumor mutations but exhibiting a high density of TLS. It will be of interest to evaluate whether the response to anti-PD1 therapy that has been linked to the mutation status of tumor cells in NSCLC patients is related to the presence of a high density of TLS in the responding patients (43). Thus, combining therapies aimed at blocking inhibitory ICP, on the one hand, and at inducing/maintaining TLS with anti-tumor ability, on the other hand, represents an exciting new therapeutic approach that could lead to the setting of a long-lasting adaptive response against tumors, promoting a curative vaccine effect in cancer patients. Of note, two reports argue in favor of a positive relationship between the development of an anti-tumor response following anti-cancer vaccination and the presence of TLS. First, it has been shown that G-VAX vaccination, based on the use of allogeneic tumor cells expressing GM-CSF, induces TLS formation in pancreatic tumors (44). In this vaccination scheme, aimed at triggering a T-cell response against mesothelin and other tumor Ags, T cells present in TLS exhibited activation markers and *in vitro* experiments demonstrated their ability to exert effector functions. A detailed analysis also showed that the G-VAX vaccination induced a Th17 polarization. The vaccinated patients had a favorable effector T cells/regulatory T cells ratio, suggesting that they could develop an anti-tumor response, even though PD1 and PD-L1 could be detected in TLS present in the tumor masses. It should be pointed out that the apparent trigger of a Th1/Th17 differentiation in TLS observed in the vaccinated patients paralleled a decrease in regulatory T cell infiltrate as well as a more prolonged survival (44). Second, Maldonado *et al*. reported that a high density of TLS has been observed in patients with cervical cancer who respond to HPV vaccine (45). Thus, TLS may also represent a useful marker of response to cancer immunotherapies. One can hypothesize that immunotherapies, initially designed to enhance an anti-tumor immune response, can make the tumor micro-environment switch from a pro-tumor to a milieu where a rapid and strong anti-tumor adaptive immunity is set up thanks to the formation of TLS.

## Conclusion

Since the discovery of the presence of TLS in human solid tumors (1), a large number of reports have highlighted the importance of these structures in the shaping of a favorable immune micro-environment capable of controlling tumor development in most cases [reviewed in Ref. (46)]. Many issues remain to be solved, such as the deciphering of the precise and sequential cellular and molecular events that lead to TLS neogenesis and their maintenance, and the role of TLS-B cells, plasma cells, and the antibodies that are being secreted locally in the control or facilitation of tumor progression. Also, if one wants to manipulate TLS to achieve an efficient long-lasting control of tumor development, we have to understand the mechanisms by which tumors can escape the immune surveillance exerted by TLS. They may include the production of immune suppressive cytokines (TGF-β, IL10), Treg recruitment and/or the action of facilitating antibodies. Overall, it is increasingly evident that TLS represent a target of choice to develop efficient anti-cancer vaccine strategies as they are the core factory where a potent adaptive anti-tumor is being generated.

## Author Contributions

Wrote/revised the paper: J-LT and M-CD-N.

## Conflict of Interest Statement

The authors declare that the research was conducted in the absence of any commercial or financial relationships that could be construed as a potential conflict of interest.
